# Case Report: From imaging to genetics: a case of congenital restrictive strabismus with SEOM expands the 22q11.2 duplication syndrome phenotype

**DOI:** 10.3389/fmed.2026.1782101

**Published:** 2026-02-23

**Authors:** Xingyuan Wei, Ruxin Gao, Renyi Xie

**Affiliations:** 1 Xiamen Eye Center and Eye Institute of Xiamen University, School of Medicine, Xiamen, China; 2 Xiamen Clinical Research Center for Eye Diseases, Xiamen, China; 3 Xiamen Key Laboratory of Ophthalmology, Xiamen, China; 4 Fujian Key Laboratory of Corneal and Ocular Surface Diseases, Xiamen, China; 5 Xiamen Key Laboratory of Cornealand and Ocular Surface Diseases, Xiamen, China; 6 Translational Medicine Institute of Xiamen Eye Center of Xiamen University, Xiamen, China

**Keywords:** 22q11.2 duplication, anomalous orbital structures, copy number variation, globe retraction, strabismus, supernumerary extraocular muscle

## Abstract

This study presents a case of restrictive strabismus with ipsilateral enophthalmos secondary to a supernumerary extraocular muscle (SEOM). Orbital MRI and posterior segment OCT provided direct imaging evidence that delineated the posterior origin of the SEOM and its mechanical traction on the globe, while also revealing concomitant hypoplasia of the medial and lateral rectus muscles. These findings together elucidate the mechanical basis of both ocular motility restriction and enophthalmos in this case. Genetic analysis revealed a pathogenic duplication in the 22q11.21 region, which—to our knowledge—is the first reported association linking this variant to SEOM-related restrictive strabismus, thereby expanding the ocular phenotypic spectrum of the 22q11.2 duplication syndrome. The discussion underscores that surgical intervention carries substantial risk due to the deep, optic-nerve-adjacent location of the SEOM and the presence of rectus muscle hypoplasia, compounded by the reportedly poorer prognosis associated with posterior SEOM (Type 3). Hence, conservative management was advised. This case highlights the central diagnostic role of MRI and offers novel insights into the etiology and individualized management of anomalous orbital structures.

## Introduction

Anomalous orbital structures (AOS) constitute a rare etiology of strabismus. Anomalies in the structure of the extraocular muscles (EOMs) or adjacent tissues are typically designated as AOS. Some of these structures can attach to the eyeball, leading to mechanical movement restrictions (1–3). Before precise imaging techniques became widely available, diagnosing restrictive strabismus caused by such structures was challenging, and confirmation of AOS relied solely on autopsy findings or observations during strabismus surgery. To systematically characterize such anomalies, Lueder et al. categorized them into three distinct types: ectopic muscles deriving from EOMs, fibrous bands located beneath the rectus muscles, and discrete anomalous muscles with posterior orbital origins and abnormal global insertions locations (4).

The pathogenic mechanism of AOS remains unclear to date, with prevailing hypotheses suggesting it may be attributed to disruptions in embryonic development or the persistence of atavistic retractor bulbi muscles (4–6). A specific and notable form of AOS is the supernumerary extraocular muscle (SEOM), where more than six muscles are present, leading to restrictive strabismus—a phenomenon first documented by Nussbaum in 1893 (7). The condition presented in this case—restrictive strabismus with enophthalmos due to a supernumerary muscle—is categorized as the third type of anomaly described above.

## Case description

**Figure 1:**
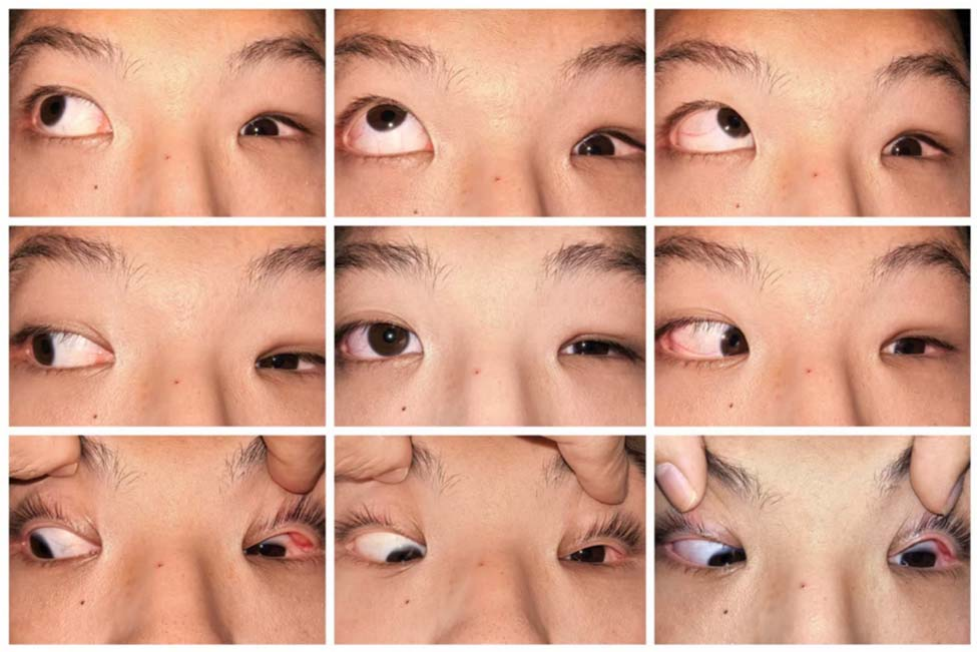
Ocular motility photographs of the patient in nine positions of gaze.

**Figure 2:**
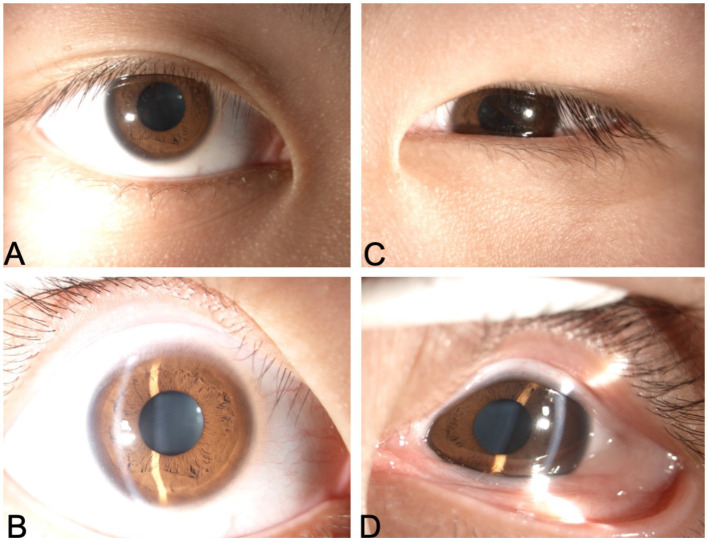
Bilateral slit-lamp photograph. **(A, B)** Right eye; **(C, D)** Left eye.

A 15-year-old boy presented to our hospital with the chief complaints of lifelong left eye enophthalmos and restricted ocular movement. It was noted that the left eye movements were restricted in all fields of gaze (Figure 1). The forced duction test is crucial for evaluating patients with ocular motility disorders. In this case, the test was performed to determine whether the limitation was due to mechanical restriction or neuromuscular paralysis. The results showed a positive forced duction test with resistance encountered in all directions, particularly notable in the inferior rectus and medial rectus directions. He was otherwise healthy. On examination, uncorrected visual acuity was 0.04 in the left eye and 0.05 in the right eye. Best-corrected visual acuity was 1.0 in the right eye with −6.00DS/−0.75DC × 180 °C and 0.3 in the left eye with −8.00DS/ −2.00DC × 180 °C. The axial length was 26.02 mm in the right eye and 25.98 mm in the left eye. Slit-lamp examination showed conjunctival accumulation in the left eye, with the conjunctival sac covering the superior and inferior limbus (Figure 2). His parents reported that the left eye abnormality had been noted since early childhood. The patient had visited multiple other hospitals previously, but no definitive cause had been identified and no treatment had been administered.

**Figure 3:**
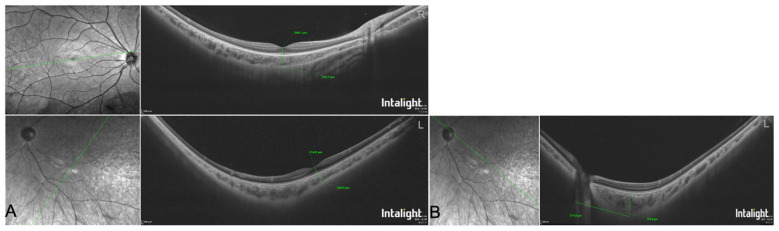
**(A)** Posterior segment OCT images of both eyes. **(B)** Significant retrobulbar traction on the left eye and the localization of the SEOM. SEOM: supernumerary extraocular muscle.

To determine the etiology, further detailed ophthalmic examinations were conducted. Regular astigmatism was identified in the right eye and irregular astigmatism in the left eye by corneal topography. Anterior segment OCT showed a significantly greater corneal thickness in the left eye (624 μm) compared to the right eye (538 μm). The morphology and density of corneal endothelial cells were normal in both eyes. Extorsion of the left fundus and a tessellated fundus in both eyes were observed on Zeiss wide-field fundus photography. Posterior segment OCT of the left eye revealed significant traction on the posterior ocular structures (Figure 3A). Localization of the SEOM, estimated to be about 3,713.4 μm inferotemporal to the optic nerve, was based on the point of greatest retrobulbar traction identified on posterior segment OCT (Figure 3B).

**Figure 4:**
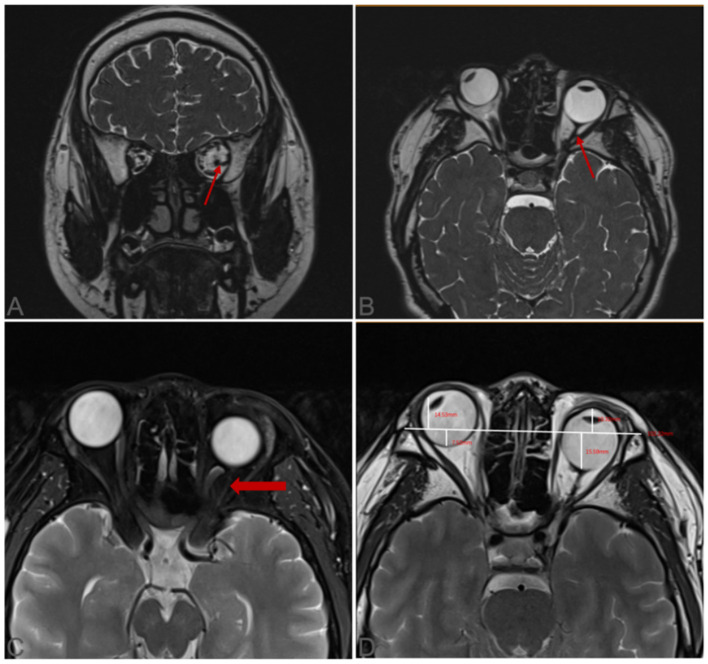
The anatomical course and insertions of the SEOM. **(A, B)** The insertion of this structure into the inner face of the lateral rectus is demonstrated by the thin arrow; **(C)** The main body of the SEOM inserted near the optic nerve and tracked toward the orbital apex is pointed by the thick arrow; **(D)** Distance of the cornea and sclera from the interzygomatic line. SEOM: supernumerary extraocular muscle.

**Figure 5:**
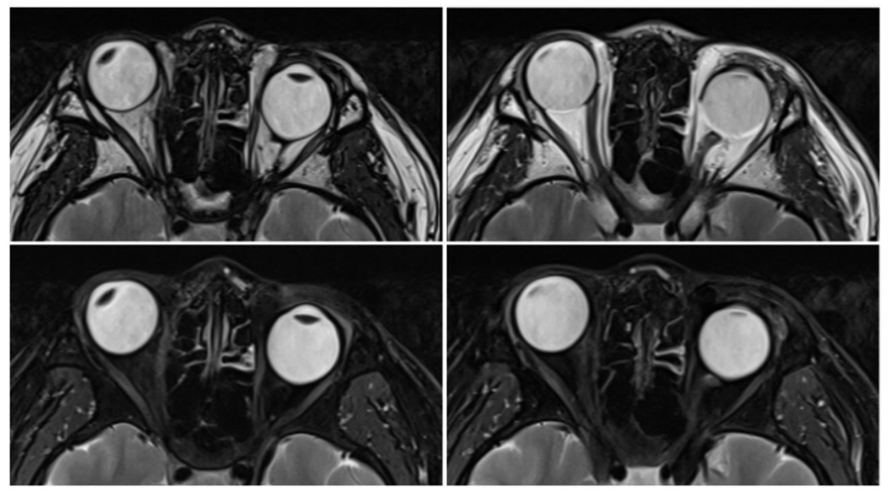
The hypoplastic medial and lateral rectus muscles in the left eye.

The SEOM location identified by these imaging findings was consistent with the orbital magnetic resonance imaging (MRI) results. Unenhanced orbital MRI demonstrated an abnormal tissue within the left intraconal space. On serial imaging slices, this tissue was localized inferotemporal to the optic nerve, medial to the lateral rectus muscle, and superior to the inferior rectus muscle (Figure 4). This tissue exhibited MRI attenuation similar to that of muscle, visually presenting as a taut muscular structure. Originating from the posterior orbital apex near the annulus of Zinn, it coursed anteriorly and inserted directly onto the posterior globe, with a secondary insertion into the lateral rectus muscle (Figure 4). Using the MRI system's built-in measurement tool, the interzygomatic line (IZL) was drawn on the T2-weighted axial image by connecting the apexes of the bilateral lateral orbital rims, serving as the reference line. The distance between IZL and cornea and the distance between IZL and posterior sclera were also measured and recorded. We obtained the following distances: the corneal–interzygomatic line distances were 14.53 mm (right eye) and 10.32 mm (left eye); the posterior sclera–interzygomatic line distances were 7.52 mm (right eye) and 15.95 mm (left eye). Through dynamic MRI observation, MRI additionally demonstrated hypoplastic development of the medial and lateral rectus muscles in the left eye (Figure 5).

To clarify the etiology, genetic analyses were performed. Initial whole-exome sequencing (WES) suggested a possible ~2.67 Mb duplication at chromosome 22q11.21 in the patient, which was classified as pathogenic (score: 1 point) according to ACMG guidelines (8). To confirm this finding and determine its origin, high-resolution genome-wide copy number variant sequencing (CNV-Seq) was subsequently conducted for the patient and both parents. No abnormal clinical findings were noted in the eyes or other systems of his parents. CNV-Seq precisely confirmed a 2.42 Mb duplicated segment at 22q11.21 (chr22:18900001_21320000) with a copy number of three in the patient, also classified as pathogenic. This CNV is located entirely within a triplosensitive region (the 22q11.2 recurrent proximal A–D region; ClinGen triplosensitivity score: 3). Unexpectedly, result of CNV-Seq of his father identified a ~1.34 Mb duplication at 22q11.23, which was categorized as a variant of uncertain significance (VUS). This CNV completely overlaps with a potential triplosensitivity region (the distal type III 22q11.2 recurrent region, spanning D–G, D–H, E–H, F–H, or F–G segments; ClinGen triplosensitivity score: 1). No clinically significant copy number variations were detected in the mother.

## Discussion

This case report presents a complex presentation of restrictive strabismus with ipsilateral enophthalmos caused by SEOM. It is the first to provide detailed ophthalmic examination data and genetic testing results for such a condition. The multifaceted analysis presented herein deepens the understanding of AOS and offers valuable insights into their etiology, diagnostic challenges, and clinical management.

SEOM is a rare and frequently overlooked cause of congenital ocular motility restriction. MRI plays a crucial role in its diagnosis and differentiation from other etiologies (1, 9–11). Historically, since the initial report by Nussbaum in 1893, most SEOM cases were incidentally discovered during exploratory strabismus surgery or postmortem examination (7). The advent of MRI has transformed this diagnostic paradigm. In the present case, orbital MRI, leveraging its superior soft-tissue resolution, clearly and non-invasively delineated the entire course of the SEOM, its spatial relationship to the optic nerve and other EOMs, and confirmed its signal characteristics consistent with muscular tissue. This study is also the first to quantify the anomaly through comparison with normative pediatric orbital structures MRI data (12). The affected eye exhibited a characteristic anatomical alteration—an increased posterior sclera-interzygomatic line distance and a decreased corneal-interzygomatic line distance—thereby providing objective imaging evidence for the mechanism of posterior globe traction with relative anterior segment retraction. Additionally, MRI clearly demonstrated hypoplastic changes in the medial and lateral rectus muscles of the affected eye, along with an aberrant connection between the SEOM and the lateral rectus muscle. The biomechanical mechanism of SEOM-induced congenital restrictive strabismus is elucidated by the collective findings presented.

Previous reports have rarely documented ocular biometry in such cases. Comprehensive imaging in this patient revealed that while the left eye had approximately 2.00 D more myopia than the right eye, the axial length difference was only 0.04 mm, a discrepancy potentially attributable to limited retrobulbar volume restricting axial elongation in the left orbit. The patient exhibited significantly greater corneal thickness in the left eye than the right, a finding not linked to genetic variants and likely resulting from developmental compensatory remodeling of corneal collagen (13, 14). The irregular astigmatism in the left eye was likely related to chronic enophthalmos and corneal compression from redundant conjunctival folds. Crucially, posterior segment OCT clearly delineated morphological alterations at the posterior globe due to traction, a finding consistent with MRI observations. When routine ophthalmic examination fails to identify the etiology of congenital restrictive strabismus, adjunctive posterior segment OCT combined with MRI analysis can enhance diagnostic accuracy. In primary eye center without MRI access, posterior segment OCT may provide pivotal initial clues to guide further investigation. This case underscores the significant diagnostic utility of posterior segment OCT in evaluating congenital ocular motility restriction. This constellation of imaging findings elucidates the biomechanical basis of the restrictive strabismus: the hypoplastic muscles combined with posterior SEOM traction create a tethering effect that restricts ocular rotation and posterior globe position, directly accounting for the observed severe motility limitation and enophthalmos.

A growing body of research in recent years has established an association between CNV and an increased risk of strabismus (15–18). The fundamental mechanism of 22q11.2 duplication syndrome involves abnormal gene dosage resulting from CNVs. To our knowledge, this study represents the first report of restrictive strabismus associated with abnormal extraocular muscles (SEOM) in relation to a proximal 22q11.2 (A–D interval) triplosensitive region duplication. This finding fills a gap in the genetic etiology of this type of strabismus and expands the ocular phenotypic spectrum of 22q11.2 duplication syndrome. Although genetic variations in the 22q11.2 region—particularly the deletion syndrome—have been well established as closely associated with ocular abnormalities (19, 20), ocular phenotypes of the duplication syndrome have been rarely reported, with no prior documentation linking it to restrictive strabismus with SEOM (21, 22). Based on the results of family CNV-seq analysis, we conclude that the genetic variant in the patient is a *de novo* variant. The duplicated region in this patient encompasses 15 OMIM-listed pathogenic protein-coding genes, including TBX1 and LZTR1 et al., and has a ClinGen triplosensitivity score of 3, indicating high sensitivity to increased gene dosage. As a key gene in this region, heterozygous loss of TBX1 has been clearly shown to disrupt craniofacial and ocular embryonic development, leading to strabismus. Therefore, we propose that the duplication in this case may cause abnormal extraocular muscle development through a similar dosage-sensitive mechanism, ultimately presenting as restrictive strabismus. This case provides the first clinical and genetic evidence linking this duplication to restrictive strabismus with SEOM and offers clues for further investigation into the role of this genomic region in ocular motor development.

The 22q11.2 microduplication syndrome demonstrates broad phenotypic variability (23, 24). The primary clinical presentation encompasses psychomotor and speech delay, intellectual disability, learning difficulties, and characteristic facial features (including ocular hypertelorism, a broad flat nasal bridge, micrognathia, ear malformations, epicanthal folds, and down-slanting palpebral fissures), along with potential manifestations such as global developmental delay, hypotonia, behavioral abnormalities, congenital heart defects, genitourinary anomalies, velopharyngeal insufficiency with or without cleft palate, epilepsy, microcephaly, craniosynostosis, ocular disorders, hearing impairment, hypernasal speech, and feeding difficulties (25–27). In the present case, only strabismus-related manifestations were observed without involvement of other organ systems, which may be attributed to the incomplete penetrance characteristic of this genetic condition. The absence of strabismus in the father may be explained by the relatively weaker association of distal region genes with extraocular muscle development and their lower sensitivity to dosage alterations (22). These are two separate, non-overlapping CNVs at different loci within 22q11.2, and that the father's variant was classified as a variant of uncertain significance, which helps explain the isolated phenotype in the proband. In summary, the phenotypic presentation in this case differs from previously reported ocular abnormalities associated with 22q11.2 CNV, indicating that strabismus may represent an isolated phenotypic manifestation of 22q11.2 CNV. This finding holds important clinical implications: for patients presenting with rare restrictive strabismus in the absence of a clear family history or other systemic abnormalities, testing for 22q11.2 region CNVs should be considered to avoid underdiagnosis of genetic disorders.

However, surgical intervention posed significant challenges. The procedure itself carried a non-negligible risk of visual impairment due to the SEOM's deep location and proximity to the optic nerve. More critically, any potential improvement in strabismus and enophthalmos would likely be limited by the patient's concomitant hypoplasia of the medial and lateral rectus muscles, compounded by the anticipated postoperative fibrosis and adhesions in the conjunctival and retrobulbar tissues. Furthermore, literature suggests outcomes are less favorable for deep, posterior SEOM (Type 3) compared to anterior case reports (3, 10). Considering the high-risk, low potential benefit profile, a conservative, non-surgical approach was therefore recommended for this patient. It is important to note that the conservative management adopted here was based on an individualized assessment of the patient's individual anatomy and risk-benefit profile. Surgery on the anteriorly located SEOMs may still be a suitable option in select cases, as noted by the cited literature.

In conclusion, SEOM should be considered in the differential diagnosis of atypical restrictive strabismus, with MRI serving as the essential tool for accurate pre-operative identification and treatment planning. This case establishes a novel association between 22q11.2 duplication and strabismus in the context of AOS, expanding the phenotypic spectrum of this syndrome and providing insights into the genetic mechanisms underlying complex ocular motility disorders.

## Data Availability

The original contributions presented in the study are included in the article/supplementary material, further inquiries can be directed to the corresponding author/s.

## References

[1] Wang D Yang YY Chang QL Ma Q Wang YD Man FY . Congenital restrictive strabismus associated with anomalous orbital structures: MRI findings and clinical Characteristics. Am J Ophthalmol. (2025) 276:195–200. doi: 10.1016/j.ajo.2025.04.013 40250744

[2] Flanders M. Restrictive strabismus: diagnosis and management. Am Orthopt J. (2014) 64:54–63. doi: 10.3368/aoj.64.1.54 25313112

[3] Goyal P Tibrewal S Lefebvre DR Ganesh S Hunter DG. Challenges in management of congenital enophthalmos due to anomalous accessory orbital extraocular muscle bands. Strabismus. (2024) 32:195–201. doi: 10.1080/09273972.2024.2344538 39072535

[4] Lueder GT. Anomalous orbital structures resulting in unusual strabismus. Surv Ophthalmol. (2002) 47:27–35. doi: 10.1016/S0039-6257(01)00285-5 11801267

[5] Whitnall SE. An instance of the retractor bulbi muscle in man. J Anat Physiol. (1911) 46:36–40. 17232909 PMC1288895

[6] Wang X Shen T Han M Yan J. Supernumerary extraocular muscle: a rare cause of atypical restrictive strabismus. Medicina (Kaunas). (2022) 58:1691. doi: 10.3390/medicina58111691 36422229 PMC9693874

[7] Nussbaum M. Vergleichend-anatomische beiträge zur kenntnis der augenmuskeln. Anat Anz. (1893) 8:208–10.

[8] Riggs ER Andersen EF Cherry AM Kantarci S Kearney H Patel A . Technical standards for the interpretation and reporting of constitutional copy-number variants: a joint consensus recommendation of the American college of medical genetics and genomics (ACMG) and the clinical genome resource (ClinGen). Genet Med. (2020) 22:245–57. doi: 10.1038/s41436-019-0686-8 31690835 PMC7313390

[9] Khitri MR Demer JL. Magnetic resonance imaging of tissues compatible with supernumerary extraocular muscles. Am J Ophthalmol. (2010) 150:925–31. doi: 10.1016/j.ajo.2010.06.007 20801423 PMC2991531

[10] Molinari A Plager D Merino P Galan MM Swaminathan M Ramasuramanian S . Accessory extraocular muscle as a cause of restrictive strabismus. Strabismus. (2016) 24:178–83. doi: 10.1080/09273972.2016.1242641 27835055

[11] Merino P de Liaño PG Ruiz Y Franco G. Atypical restrictive strabismus secondary to an anomalous orbital structure: differential diagnosis. Strabismus. (2012) 20:162–5. doi: 10.3109/09273972.2012.702326 23211141

[12] Almus E Sen Akova B Ozer H Fitoz S. Orbital structures in the pediatric age group: a normative assessment using magnetic resonance imaging. Eur J Radiol. (2022) 154:110418. doi: 10.1016/j.ejrad.2022.110418 35772338

[13] Tidu A Schanne-Klein MC Borderie VM. Development, structure, and bioengineering of the human corneal stroma: a review of collagen-based implants. Exp Eye Res. (2020) 200:108256. doi: 10.1016/j.exer.2020.108256 32971095

[14] Bergmanson JPG Burns AR Walker MK. Central versus peripheral thickness in the human cornea explained. Cont Lens Anterior Eye. (2024) 47:102165. doi: 10.1016/j.clae.2024.102165 38589268

[15] Sanchez MM Whitman MC. Genetics of strabismus. Front Ophthalmol (Lausanne). (2023) 3:1233866. doi: 10.3389/fopht.2023.1233866 38500555 PMC10947184

[16] Viola Lee KA Whitman MC. Rare and common genomic copy number variants associated with strabismus and amblyopia in the all of us research program. medRxiv [Preprint]. (2025). doi: 10.1101/2025.11.03.25339429 42411868 PMC13361834

[17] Whitman MC Di Gioia SA Chan WM Gelber A Pratt BM Bell JL . Recurrent rare copy number variants increase risk for esotropia. Invest Ophthalmol Vis Sci. (2020) 61:22. doi: 10.1167/iovs.61.10.22 32780866 PMC7443120

[18] Martinez Sanchez M Chan WM MacKinnon SE Barry B Hunter DG Engle EC . Presence of copy number variants associated with esotropia in patients with exotropia. JAMA Ophthalmol. (2024) 142:243–47. doi: 10.1001/jamaophthalmol.2023.6782 38358749 PMC10870223

[19] Szczawińska-Popłonyk A Schwartzmann E Chmara Z Głukowska A Krysa T Majchrzycki M . Chromosome 22q11.2 deletion syndrome: a comprehensive review of molecular genetics in the context of multidisciplinary clinical approach. Int J Mol Sci. (2023) 24:8317. doi: 10.3390/ijms24098317 37176024 PMC10179617

[20] von Scheibler E van der Valk Bouman ES Nuijts MA Bauer NJC Berendschot T Vermeltfoort P . Ocular findings in 22q11.2 deletion syndrome: a systematic literature review and results of a Dutch multicenter study. Am J Med Genet A. (2022) 188:569–78. doi: 10.1002/ajmg.a.62556 34773366 PMC9298823

[21] Cordovez JA Capasso J Lingao MD Sadagopan KA Spaeth GL Wasserman BN . Ocular manifestations of 22q11.2 microduplication. Ophthalmology. (2014) 121:392–98. doi: 10.1016/j.ophtha.2013.06.040 23972321

[22] Xue J Shen R Xie M Liu Y Zhang Y Gong L . 22q11.2 recurrent copy number variation-related syndrome: a retrospective analysis of our own microarray cohort and a systematic clinical overview of ClinGen curation. Transl Pediatr. (2021) 10:3273–81. doi: 10.21037/tp-21-560 35070841 PMC8753460

[23] Wentzel C Fernström M Ohrner Y Annerén G Thuresson AC. Clinical variability of the 22q11.2 duplication syndrome. Eur J Med Genet. (2008) 51:501–10. doi: 10.1016/j.ejmg.2008.07.005 18707033

[24] Rosenfeld JA Coe BP Eichler EE Cuckle H Shaffer LG. Estimates of penetrance for recurrent pathogenic copy-number variations. Genet Med. (2013) 15:478–81. doi: 10.1038/gim.2012.164 23258348 PMC3664238

[25] Dell'Edera D Allegretti A Ventura M Mercuri L Mitidieri A Cuscianna G . Mayer-Rokitansky-Küster-Hauser syndrome with 22q11.21 microduplication: a case report. J Med Case Rep. (2021) 15:208. doi: 10.1186/s13256-021-02716-6 33883018 PMC8058992

[26] Bartik LE Hughes SS Tracy M Feldt MM Zhang L Arganbright J . 22q11.2 duplications: Expanding the clinical presentation. Am J Med Genet A. (2022) 188:779–87. doi: 10.1002/ajmg.a.62577 34845825

[27] Purow J Waidner L Ale H. Review of the pathophysiology and clinical manifestations of 22q11.2 deletion and duplication syndromes. Clin Rev Allergy Immunol. (2025) 68:23. doi: 10.1007/s12016-025-09035-4 40038168

